# HIV-related Stigma among People with HIV in Denmark and its Association with Psychosocial and Sexual Health: a cross-sectional Nationwide Study

**DOI:** 10.1007/s10461-025-04806-8

**Published:** 2025-06-14

**Authors:** Ditte Scofield, Nina Weis, Alessandra Meddis, Merete Storgaard, Gitte Pedersen, Isik S. Johansen, Terese L. Katzenstein, Christian Graugaard, Lars H. Omland, Morten Frisch, Ellen Moseholm

**Affiliations:** 1 Department of Infectious Diseases, Copenhagen University Hospital, Hvidovre, Denmark; 2 Department of Clinical Medicine, Faculty of Health and Medical Sciences, University of Copenhagen, Copenhagen, Denmark; 3 Department of Public Health, University of Copenhagen, Copenhagen, Denmark; 4 Department of Infectious Diseases, Aarhus University Hospital, Aarhus, Denmark; 5 Department of Infectious Diseases, Aalborg University Hospital, Aalborg, Denmark; 6 Department of Infectious Diseases, Odense University Hospital, Odense, Denmark; 7 Department of Infectious Diseases, Copenhagen University Hospital, Rigshospitalet, Copenhagen, Denmark; 8 Department of Clinical Medicine, Center for Sexology Research, Aalborg University, Aalborg, Denmark; 9 Department of Bacteria, Parasites and Fungi, Research Unit for Reproductive Microbiology, Statens Serum Institut, Copenhagen, Denmark; 10 Department of Epidemiology Research, Project SEXUS Group, Statens Serum Institut, Copenhagen, Denmark

**Keywords:** HIV, HIV-related stigma, Sexual health, Mental health

## Abstract

HIV-related stigma remains a major barrier to improving the lives of people with HIV (PWH) and curbing the epidemic globally. This cross-sectional study assessed HIV-related stigma, based on The HIV Stigma Framework, among PWH in Denmark, and examined its potential associations with sociodemographic characteristics, as well as with HIV-related, psychosocial and sexual health measures. Data from 630 participants (486 men and 144 women) in the SHARE study—a nationwide survey on psychosocial and sexual health among PWH in Denmark conducted from 2021 to 2022—were analysed using sex-stratified linear and logistic regression models adjusted for relevant confounders. Results showed that anticipated stigma was the most commonly reported stigma mechanism, with 68% of men and 77% of women expressing high levels of concern about HIV status sharing. In contrast, enacted stigma was reported by 9% of men and 22% of women. The multivariate regression analyses revealed that higher stigma levels were associated with originating from low- or middle-income countries and with a history of mental health problems, whereas non-heterosexual identity and a longer time since HIV diagnosis were linked to lower stigma. In both men and women, stigma was significantly associated with fewer close friends, loneliness, anxiety and reduced sexual desire. Among men, stigma was also significantly associated with sharing one’s HIV status with fewer people, depression, suicidal tendencies and erectile dysfunction. In conclusion, HIV-related stigma, notably stigma related to anticipated stigma, remains prevalent among PWH in Denmark and it is significantly associated with a range of psychosocial and sexual challenges.

**Supplementary Information:**

The online version contains supplementary material available at 10.1007/s10461-025-04806-8.

## Background

Significant advances in the treatment of human immunodeficiency virus (HIV) infection have transformed HIV from a life-threatening disease into a long-term, chronic condition with near-normal life expectancies for individuals achieving viral suppression [1, 2]. Despite this progress, marked health disparities persist in several areas, including sexual [3, 4] and mental health [5, 6], rendering people with HIV (PWH) susceptible to a diminished quality of life.

When HIV emerged more than four decades ago, limited understanding of the virus existed. The devastating morbidity and mortality associated with an HIV diagnosis, and the fact that it initially affected an already stigmatised community of men who had sex with men, led to significant societal stigmatisation and discrimination against PWH [7]. Despite substantial progress since then, PWH continue to experience high levels of stigma [8, 9], exceeding those encountered by individuals with other chronic medical conditions [10]. Notably, the Joint United Nations Programme on HIV/AIDS (UNAIDS) has identified HIV-related stigma as the primary barrier to halting the global HIV epidemic [11], as it prevents people from adopting preventive measures, getting tested and accessing appropriate treatment once diagnosed.

Stigmatisation is conceptualised as a social process in which individuals or groups possessing a characteristic viewed negatively by society, such as living with HIV, are subjected to discrimination, exclusion and prejudice. These processes lead to loss of status and unequal treatment, and are enabled by broader social inequalities, including differences in influence, resources or respect [12]. HIV-related stigma specifically operates through multiple mechanisms, as described by Earnshaw and Chaudoir in the HIV Stigma Framework [13]: *enacted stigma* (direct experiences of discrimination); *anticipated stigma* (expectations of discrimination or rejection); and *internalised stigma* (adopting negative self-beliefs based on one’s HIV status). This framework illustrates the diverse ways in which HIV-related stigma can affect individuals with HIV. HIV-related stigma has been identified as the primary source of psychological distress in PWH [14] and has been associated with a spectrum of adverse health outcomes including depression and anxiety [15, 16], poor physical health outcomes [17], low social support [15, 17], problems with antiretroviral therapy (ART) adherence and disengagement from HIV care [7, 18, 19]. A recent meta-analysis revealed stable and consistent associations between stigma, reduced quality of life and diminished well-being among PWH [14] over the past two decades, despite significant advances in HIV treatment.

However, understanding stigma purely from this subjective HIV stigma framework is insufficient, as it overlooks critical intersectional and structural dimensions of stigma affecting PWH. Meyer’s minority stress theory [20] offers valuable insights by highlighting how social stigma, prejudice and discrimination specifically directed towards minority groups, such as sexual and ethnic minorities, exacerbate health disparities through chronic, socially-based stress processes. According to Meyer, minority stress includes external experiences (e.g., discrimination), internal experiences (e.g., internalised stigma, concealment or fear of rejection) and coping resources like social support and community involvement [20].

Intersectional stigma further complicates the experiences of PWH, particularly among sexual and ethnic minority groups who already experience societal marginalisation [21–23]. For example, Gruszczyńska & Rzeszutek [21] demonstrated how multiple intersecting minority identities (such as living with HIV and belonging to a sexual or ethnic minority) amplify experiences of stigma, resulting in intensified psychosocial distress and poorer health outcomes. Similarly, Cramer et al. [22] found that internalised stigma, both sexual orientation-related and HIV-specific, strongly predicts negative emotional states and overall reduced affective well-being among sexual minority PWH. Additionally, structural stigma, as discussed by Hatzenbuehler et al. [23], influences health outcomes through discriminatory societal attitudes and systemic barriers, affecting substance use, HIV risk behaviours and mental health.

Denmark’s provision of taxpayer-funded healthcare, its high success rates in diagnosing and suppressing HIV [24], and the country’s relatively open public discourse on sexual matters [25] create an environment conducive to optimal psychosocial and sexual health for PWH, as well as the potential elimination of HIV-related stigma at both individual and societal levels. However, despite these favourable conditions, our previous studies have identified a disproportionate burden of psychosocial and sexual health challenges among women [26], men who have sex with men [27], and people aged 50 and above [28] living with HIV in Denmark, suggesting that stigma may continue to influence health even in seemingly optimal contexts.

On this basis, the present study aimed, first, to investigate the extent of HIV-related stigma experienced by PWH in Denmark and the characteristics associated with experiencing stigma. Second, building on the findings of previous studies [26–28], to explore potential associations between HIV-related stigma and indicators of HIV self-management, psychosocial and sexual health in order to evaluate whether stigma may be a significant factor contributing to these health disparities.

## Methods

### Setting

There are approximately 5,700 people diagnosed with HIV in Denmark with an additional 250 estimated to be undiagnosed [29]. Men who have sex with men account for 60% of the population, 25% are women, and approximately 40% were born outside of Denmark [29, 30]. Specialised infectious disease clinics offer HIV management where the majority of PWH attend care once or twice a year. Denmark has consistently met the UNAIDS’ global HIV treatment targets [11, 30].

### Study Design

This cross-sectional questionnaire-based study used data from the nationwide survey study “*Perception of psychosocial*,* sexual*,* and reproductive health among people living with and without HIV in Denmark - the SHARE study”*. The SHARE study’s aim was to examine sexual, psychosocial and reproductive health among all PWH in Denmark. PWH were recruited by healthcare professionals during scheduled clinic appointments between March 2021 and February 2022. Recruitment took place at the five largest university hospital infectious disease clinics in Denmark, including Aarhus, Aalborg, Odense and the Copenhagen University Hospitals, Hvidovre and Rigshospitalet. No financial compensation was provided for participation in the study.

### Population

The eligibility criteria included age ≥ 18 years, an HIV diagnosis according to the ICD-10 criteria and the ability to read and understand Danish or English. In total, 1,000 PWH consented to participate during the recruitment process, 652 initiated the survey and 623 completed the full study questionnaire. In the analyses of the present study, only data from cisgendered PWH were included. Data on PWH who declined participation were not recorded in the recruitment process. Hence, in an effort to assess the representativeness of our study population, clinical HIV and certain sociodemographic characteristics were compared to data from the Danish HIV Cohort Study, a national cohort of all PWH who attend HIV-related care in Denmark [31].

### Data Collection

A link to an electronic questionnaire was sent to the participants shortly after recruitment. The questionnaire was available in Danish and English and had been pilot tested and validated through interviews with a diverse group of PWH. It took approximately 25–30 min to complete.

### Variables

The study questionnaire consisted of individual items as well as standardised and validated patient-reported outcome measures related to HIV, psychosocial and sexual health. The questionnaire was in part inspired by the large, population-based Danish cohort study Project SEXUS [32].

*The 12-item short version of the HIV Stigma Scale* (HSS-12) was used to measure overall HIV-related stigma (from here on referred to as stigma) [33, 34]. The scale includes four stigma domains: *Personalised stigma*, representing enacted stigma; *Concerns about sharing status* and *Concerns with public attitudes*, both proxies for anticipated stigma; and *Negative self-image*, related to internalised stigma, all in accordance to the HIV Stigma Framework [13]. The HSS-12 includes a total of 12 items with the four stigma domains represented in four subscales with three items in each. Responses are scored on a 4-point Likert scale ranging from ‘strongly disagree’ (1) to ‘strongly agree’ (4). Domain scores (range 3–12) and a total score (range 12–48) were calculated with higher scores indicating higher levels of stigma. The HSS-12 has been shown to be a reliable and valid measure of stigma [35] and has been validated in a Danish population [36]. In accordance with previous studies [37, 38], a domain cut-off score of > 7.5, indicating high levels of stigma, was also used.

The validated Terry Beirn Community Programs for Clinical Research on AIDS *Antiretroviral Medication Adherence Self-Report Form* was used to assess ART adherence [39]. The participants were asked to complete the following: “During the last 7 days I took…” with one of the following options: ‘all’, ‘most’, ‘about one-half’, ‘very few’, or ‘none’ “…of my pills”. Responses were dichotomised into optimal (all pills = 100%) or suboptimal (< 100%) adherence in the past 7 days.

HIV clinic attendance was assessed using the question: “In the past 12 months, have you missed any appointments related to the treatment of your HIV, e.g., at the hospital or with the doctor” with response options including ‘no’, ‘yes, once’, ‘yes, twice’, ‘yes, three times or more’. Responses were dichotomised into complete (100%) or incomplete (< 100%) attendance in the past 12 months.

HIV status sharing was assessed using an item from the *Danish Living Condition Survey* [40]: “How many people have you told that you are living with HIV? - excluding healthcare staff and HIV-counselors” with the response options ‘none’, ‘1–2 people’, ‘3–5 people’, ‘6–10 people’ and ‘more than 10 people’. Responses were dichotomised into two categories: ‘0–2 people’ and ‘≥3 people’.

To assess overall HIV self-management, information related to ART adherence and HIV clinic attendance was combined. PWH reporting both optimal (100%) ART adherence and complete (100%) HIV clinic attendance were categorised as having ‘optimal’ HIV self-management, whereas PWH with suboptimal (< 100%) ART adherence or incomplete (< 100%) HIV clinic attendance, or both, were considered to have ‘suboptimal’ HIV self-management.

The 7-item *Generalized Anxiety Disorder Screener* (GAD-7) was used as a measure of general anxiety symptoms experienced in the last two weeks [41]. The scale consists of seven items, each scored on a 4-point Likert scale (0–3). Total scores range from 0 to 21 with higher scores reflecting greater symptom severity, and scores ≥ 5 are indicative of a mild-to-severe anxiety disorder.

The 2-item *Patient Health Questionnaire* (PHQ-2) consists of two items assessing symptoms of depression in the preceding two weeks [42]. Each item is scored on a 4-point Likert scale (0–3) with total scores ranging from 0 to 6. Higher scores reflect greater symptom severity with scores of ≥ 3 considered indicative of possible clinical depression.

The 6-item *Female Sexual Function Index* (FSFI-6) is a validated scale used to assess symptoms of sexual dysfunction among sexually active women in the last four weeks [43]. Each item is scored on a 5-point Likert scale (1–5), with total scores ranging from 6 to 30. Lower scores indicate poorer sexual functioning with total scores ≤ 19 considered indicative of sexual dysfunction.

The validated 5-item *International Index of Erectile Function* (IIEF-5) was used to assess symptoms of erectile dysfunction in men based on experiences during sexual intercourse in the last four weeks [44]. To adapt the scale to men with male partners, the phrase “sexual intercourse” was replaced with “anal sex (with you as the ‘active’ partner)” in items 3–5 for this group, as reported elsewhere [45, 46]. Each of the five items is scored on a 5-point Likert scale (1–5) with a total score ranging from 5 to 25. The scores were categorised into ‘normal erectile function’ (scores 22–25) and ‘erectile dysfunction’ (scores 5–21).

Clinical HIV data were collected at the time of study enrolment and included year of HIV diagnosis, mode of acquisition, ART treatment regimen, CD4 cell count and HIV RNA viral load.

### Statistical Analyses

Categorical variables were summarised with count and proportion (n, %), and continuous variables with median value and interquartile range (IQR) or mean value with standard deviation (SD).

To assess the representativeness of our study population, the sex ratio of the study population, as well as the distributions of age, time since HIV diagnosis, country of birth, CD4 cell counts and HIV RNA viral loads were compared to those of the total population of PWH in Denmark, using Pearson’s Chi-squared test or Mood’s median test, as appropriate.

In all the statistical analyses, stigma as measured by the HSS-12 scale was treated as a continuous variable. A total HSS-12 score and sum scores for each of the four stigma domains were calculated.

Given our two distinct objectives, firstly, identifying characteristics associated with stigma and secondly, exploring how stigma may influence various health outcomes, the stigma variable was treated either as the dependent or independent variable accordingly, as detailed below.

To identify characteristics associated with stigma, univariate and multivariate linear regression analyses were conducted. The univariate models included covariates that we had previously identified as differing between PWH and people without HIV in Denmark [26, 27]. These included sex, age, country of birth, relationship status, sexual identity, employment status, education, financial situation, history of physical health problems, history of mental health problems, smoking, alcohol, recreational drug use and time since HIV diagnosis, as detailed in the footnote of Table S1 (Online Resource 1).

Based on existing literature and chosen a priori, the following covariates were included in the multivariate linear regression analysis: sex (man/woman), age (continuous, in one year intervals), country of birth (born in high/low- or middle-income country, classified according to the World Bank grading system [47]), sexual identity (heterosexual/non-heterosexual), education (≤/>10 years), history of treatment for mental health problems (never/ever), history of recreational drug use (cannabis, euphoriant or hallucinogenic drugs - never/ever) and time since HIV diagnosis (continuous, in one year intervals).

Sex-stratified logistic regression models provided odds ratios for associations between stigma and HIV-specific, psychosocial and sexual health characteristics. The results of the logistic regression models are presented as adjusted odds ratios (aORs) with 95% confidence intervals (CIs). In the analysis of HIV-specific characteristics, aORs were adjusted for age (18–29, 30–39, 40–49, 50–59 and ≥ 60 years), country of birth, sexual identity and time since HIV diagnosis. The aORs presented from the psychosocial and sexual health analyses were adjusted for age, sexual identity, country of birth, relationship status (married or in a steady relationship – yes/no), financial situation (difficulties paying bills in the last year – yes/no), time since HIV diagnosis, history of treatment for mental health problems and history of recreational drug use.

All presented *p* values are two-sided with a significance level of 0.05. The statistical analyses were performed in *R* version 4.0.2.

### Ethical Considerations

The SHARE study was approved by the Danish Data Protection Agency (approval no. P2021-167). Approval from the Danish National Committee on Health Ethics was not required as no biomedical interventions were included in the study. All participants provided informed consent before data collection.

## Results

In total, data from 486 men and 144 women with HIV were included in the analyses and their sociodemographic, health-related and HIV characteristics are shown in Table 1. We observed a median age of 55 years [IQR 47–62] for men and 49 years [IQR 42–55] for women, 52% of women were born outside Denmark, and 77% of men identified as non-heterosexual. The participants had lived with HIV for a considerable period of time (median men: 18 years [IQR 12–26]; women: 21 years [IQR 12–26]), and > 97% had HIV RNA levels below 50 copies/ml. Over 90% reported optimal adherence to ART in the past seven days and had attended all HIV-related clinic appointments in the last year. About one-in-five reported that they had shared their HIV status with no one or only one or two people.

**Table 1:** Sociodemographic, health-related and HIV-specific characteristics of 486 men and 144 women with HIV in Denmark (*SHARE* study, 2021–2022)

	Men (*n*, %)	Women (*n*, %)
Age		
Median [IQR]	55 [47;62]	49 [42;55]
18–39 years	46 (9.5)	26 (18.1)
40–59 years	283 (58.2)	95 (66.0)
≥ 60 years	157 (32.2)	23 (16.0)
Country of birth^a^		
Born in Denmark	408 (84.0)	68 (47.6)
Born in other high-income country	42 (8.6)	7 (4.9)
Born in low- or middle-income country	36 (7.4)	68 (47.6)
Relationship status		
Married/in steady relationship	289 (59.5)	90 (62.5)
Single	186 (38.3)	52 (36.1)
Sexual identity		
Heterosexual	108 (22.4)	118 (82.5)
Homo- or bisexual	368 (76.3)	6 (4.2)
Other^b^	6 (1.2)	19 (13.3)
Employment status		
Employed/full-time student	321 (66.0)	106 (73.6)
Unemployed	25 (5.1)	8 (5.6)
Other^c^	140 (28.8)	30 (20.8)
Education		
Short (≤ 10 years)	58 (11.9)	28 (19.4)
Medium (11–14 years)	344 (70.8)	94 (65.3)
Long (≥ 15 years)	82 (16.9)	21 (14.6)
Financial difficulties		
Difficulties paying bills in the last year		
No	421 (86.6)	121 (84.0)
Yes	54 (13.2)	23 (16.0)
History of physical health problems^d^		
No	304 (62.6)	97 (63.2)
Yes	174 (35.8)	43 (34.7)
History of mental health problems^e^		
No	291 (59.9)	91 (67.4)
Yes	191 (39.3)	50 (29.9)
Substance use		
Current smoker		
No	373 (76.7)	121 (84.0)
Yes	111 (22.8)	23 (16.0)
Alcohol intake		
≤ 7 units per week	336 (69.3)	124 (86.1)
> 7 units per week	144 (29.7)	13 (9.0)
Recreational drug use^f^		
Never	200 (41.2)	99 (80.5)
More than 12 months ago	235 (48.4)	21 (17.1)
Within the last year	45 (9.3)	3 (2.4)
HIV characteristics		
Time since HIV diagnosis, median [IQR]	18 [12;26]	21 [12;26]
Mode of HIV acquisition		
Sexual	475 (97.7)	129 (89.6)
Other^g^	11 (2.3)	15 (10.4)
CD4 count > 350 cells/µl	450 (92.6)	135 (93.8)
HIV RNA < 50 copies/ml	478 (98.4)	140 (97.2)
Self-reported adherence to antiretroviral therapy in the last seven days		
Optimal (100%)	471 (96.6)	136 (94.4)
Suboptimal (< 100%)	14 (2.9)	7 (4.9)
Self-reported adherence to HIV-related clinic appointments in the last year		
Complete attendance	451 (92.8)	132 (91.7)
Missed one or more appointments	35 (7.2)	12 (8.3)
Shared HIV status with		
0–2 people	103 (21.2)	32 (22.2)
≥ 3 people	375 (77.2)	109 (75.7)

Interquartile range [IQR]; Antiretroviral therapy (ART)

Numbers do not always sum up to 144 for women or 486 for men as those who responded “I do not know” were excluded

^a^Countries are categorised according to the World Bank economic status index

^b^Includes asexual, undecided or unplaceable sexual identities

^c^Includes in early retirement, retired, on long-term sick leaves, stay-at-home parent and other unspecified statuses

^d^Answers to the question “Have you ever been treated by a doctor for a long-lasting or severe physical disease (other than HIV)?”

^e^Answers to the question “Have you ever received treatment by a doctor, psychologist or similar professional for a mental health problem?”

^f^Cannabis or euphoriant or hallucinogenic drugs (e.g., speed, cocaine, ecstasy, mushrooms, LSD or khat)

^g^Via IV drug use, blood transfusion, vertical transmission, other or unknown

When comparing data from the study participants with data from the general population of PWH in Denmark, as registered in the Danish HIV Cohort Study [24], there was no statistically significant difference in age. However, a greater proportion of the study population had been born in high-income countries compared to the general population of PWH (men: 93% vs. 84% *p* < 0.001; women: 53% vs. 40% *p* < 0.01), and, among men, more had HIV RNA loads < 50 copies/ml (98% vs. 95% *p* < 0.001). The study population had a slightly smaller proportion of women compared to the general population of PWH (23% vs. 27% *p* = 0.03).

### Stigma Scores

Overall, the total HSS-12 mean scores were 25.6 (SD ± 7.1) for men and 28.3 (SD ± 7.5) for women. The highest scores were observed in the stigma domain *Concerns about sharing status*, with mean scores for men 8.6 (SD ± 2.6) and 9.1 (SD ± 2.5) for women. In this domain, 68% of men and 77% of women had scores > 7.5, indicating high levels of stigma (Table 2). This was followed by the domain *Concerns about public attitudes*, where men had mean scores of 6.4 (SD ± 2.2) and women had mean scores of 7.0 (SD ± 2.3). In this domain, 26% of men and 38% of women had scores > 7.5. In the *Negative self-image* domain, men had a mean score of 5.6 (SD ± 2.5) and women had a mean score of 6.3 (SD ± 2.6), with 20% of men and 30% of women scoring > 7.5. The lowest scores were seen in the *Personalised stigma* domain, where men had mean scores of 5.0 (SD ± 2.0) and women had mean scores of 5.8 (SD ± 2.2), and 9% of men and 22% of women had scores > 7.5. Across all domains, women consistently scored higher than men.

**Table 2:** Overall HSS-12 mean scores and proportions reporting high levels (domain scores > 7.5) among people with HIV in Denmark (*SHARE* study, 2021–2022)

	Men (*n* = 486)		Women (*n* = 144)	
		Domain scores > 7.5		Domain scores > 7.5
	Mean, SD	n, %	Mean, SD	n, %
Total HSS-12 score	25.6 (7.1)		28.3 (7.5)	
Personalised stigma	5.0 (2.0)	45 (9.3)	5.8 (2.2)	31 (21.5)
Concerns about sharing status	8.6 (2.6)	332 (68.3)	9.1 (2.5)	111 (77.1)
Concerns about public attitudes	6.4 (2.2)	128 (26.3)	7.0 (2.3)	55 (38.2)
Negative self-image	5.6 (2.5)	99 (20.4)	6.3 (2.6)	44 (30.1)

Standard deviations (SD); The 12-item short version of The HIV Stigma Scale (HSS-12)

### Characteristics Associated with Stigma

Univariate linear regression analyses showed that sex, age, country of birth, sexual identity, employment status, education, history of mental health problems, recreational drug use and time since HIV diagnosis were all statistically significantly associated with stigma in more than one domain (Table S1, Online Resource 1). Multivariate linear regression analyses identified country of birth and history of mental health problems as factors associated with stigma in both the total HSS-12 score and in three out of four domains (Table 3). Specifically, originating from a low- or middle-income country compared to a high-income country was associated with an average increase in the total stigma scores of 2.63 points (*p* < 0.01). Similarly, a history of mental health problems was associated with an average increase in total stigma score of 1.86 points (*p* < 0.01), time since HIV diagnosis was significantly inversely associated with total stigma scores (-0.10 points, *p* < 0.01), as well as scores in the *Concerns about sharing status* (-0.05 points, *p* < 0.001) and *Negative self-image* (-0.03 points, *p* < 0.05) domains. Identifying as non-heterosexual was significantly inversely associated with *Concerns about sharing status* (-0.68 points, *p* < 0.01) and *Concerns about public attitudes* (-0.05 points, *p* < 0.05). Age was only significantly associated with lower scores in *Concerns about public attitudes* (-0.02 points, *p* < 0.01). Sex and a history of recreational drug use did not remain significantly associated with stigma in the multivariate analysis when adjusted for confounders.

**Table 3:** Multivariate linear regression analysis of factors associated with HIV-related stigma among people with HIV in Denmark (*SHARE* study, 2021–2022)

	Total HSS-12 score		Personalised stigma		Concerns about sharing status		Concerns about public attitudes		Negative self-image	
	β	[95% CI]	β	[95% CI]	β	[95% CI]	β	[95% CI]	β	[95% CI]
Sex										
Man	REF		REF		REF		REF		REF	
Woman	0.572	[− 1.180; 2.324]	0.499	[− 0.008; 1.005]	0.290	[− 0.912; 0.332]	0.012	[− 0.518; 0.541]	0.352	[− 0.218; 0.922]
Age										
Per 1-year increase	− 0.036	[− 0.095; 0.024]	0.004	[− 0.013; 0.021]	− 0.002	[− 0.023; 0.019]	− 0.024**	[− 0.042; − 0.006]	− 0.013	[− 0.033; 0.006]
Country of birth^a^										
Born in high-income country	REF		REF		REF		REF		REF	
Born in low- or middle-income country	2.629**	[0.828; 4.430]	0.662*	[0.143; 1.182]	0.719*	[0.082; 1.357]	0.718**	[0.175; 1.261]	0.531	[− 0.054; 1.115]
Sexual identity										
Heterosexual	REF		REF		REF		REF		REF	
Non-heterosexual	− 1.318	[− 2.717; 0.0812]	− 0.133	[− 0.537; 0.270]	− 0.678**	[− 1.174; − 0.183]	− 0.446*	[− 0.868; − 0.024]	− 0.061	[− 0.515; 0.394]
Education										
Longer (> 10 years)	REF		REF		REF		REF		REF	
Shorter (≤ 10 years)	0.683	[− 1.003; 2.369]	0.266	[− 0.220; 0.753]	− 0.082	[− 0.679; 0.515]	− 0.051	[− 0.559; 0.457]	0.550*	[0.002; 1.097]
History of mental health problems^b^										
No	REF		REF		REF		REF		REF	
Yes	1.861**	[0.689; 3.033]	0.459**	[0.1208; 0.797]	0.094	[− 0.321; 0.509]	0.586**	[0.232; 0.939]	0.723***	[0.342; 1.103]
Recreational drug use^c^										
Never	REF		REF		REF		REF		REF	
Ever	− 0.564	[− 1.776; 0.648]	− 0.273	[− 0.623; 0.076]	− 0.314	[− 0.743; 0.115]	0.006	[− 0.360; 0.371]	0.017	[− 0.376; 0.411]
Time since HIV diagnosis										
Per 1-year increase	− 0.101**	[− 0.168; − 0.034]	− 0.006	[− 0.026; 0.013]	− 0.048***	[− 0.072; − 0.024]	− 0.020	[− 0.040; 0.001]	− 0.027*	[− 0.048; − 0.005]

The 12-item short version of The HIV Stigma Scale (HSS-12); Confidence intervals (CI); Reference group (REF); *P* values: ***p < 0.001; **p < 0.01; *p < 0.05. *Interpretation*: The β value reflects the change in HSS-12 score for each category, or per 1-year increase, of the covariate compared to its reference group. This analysis was done on 599 participants who responded to items related to all the included covariates, excluding missing or ‘I do not know’ responses

^a^Indexed according to The World Bank world development indicators

^b^Assessed by the question “Have you ever received treatment by a doctor, psychologist or similar professional for a mental health problem?”

^c^Includes cannabis, euphoriant or hallucinogenic drugs

### Association between Stigma and HIV-specific, Psychosocial and Sexual Health Characteristics

No significant associations were observed between stigma and HIV self-management in either sex (Table 4).

**Table 4:** Associations between HIV-related stigma and HIV-specific characteristics among people with HIV in Denmark (*SHARE* study, 2021–2022)

	HIV self-management^a^		HIV status sharing	
	Suboptimal vs. optimal (REF)		Shared status with 0–2 people vs. ≥3 people (REF)	
	aOR^b^ [95%CI]		aOR^b^ [95%CI]	
	Men (*n* = 480)	Women (*n* = 140)	Men (*n* = 472)	Women (*n* = 137)
Total HSS-12 score	0.99 [0.95;1.04]	0.99 [0.92;1.06]	1.12 [1.08;1.17]	0.96 [0.89;1.03]
Personalised stigma	1.04 [0.90;1.21]	1.00 [0.78;1.28]	1.28 [1.14;1.44]	0.94 [0.76;1.17]
Concerns about sharing status	0.91 [0.80;1.02]	1.01 [0.79;1.28]	1.84 [1.57;2.15]	1.01 [0.80;1.29]
Concerns about public attitude	0.97 [0.83;1.12]	0.83 [0.64;1.07]	1.27 [1.13;1.43]	0.92 [0.73;1.14]
Negative self-image	1.06 [0.93;1.22]	1.02 [0.82;1.27]	1.17 [1.05;1.30]	0.76 [0.59;0.98]

Reference group (REF); Adjusted odds ratio (aOR); Confidence intervals (CI); The 12-item short version of The HIV Stigma Scale (HSS-12)

*Interpretation*: The aOR reflects the change in odds of the examined HIV-specific characteristics per one unit increase in the HSS-12 score. Missing or ‘I do not know’ responses are not included in the OR calculations, hence the numbers do not add up 486 for men and 144 for women

^a^Optimal HIV self-management implies both optimal (100%) ART adherence in the last seven days and complete (100%) attendance to all HIV-related medical appointments in the last year

^b^Adjusted for age, country of birth, sexual identity and time since HIV diagnosis

In men, all stigma domains were significantly associated with higher odds of having shared one’s HIV status with no one or only 1–2 people. For example, in men, in the domain *Concerns about public attitudes*, the aOR was 1.27 [95%CI 1.13–1.43], showing that for each unit increase in stigma score related to concerns about public attitudes, the odds of having shared one’s HIV status with no one or only 1–2 people were 27% higher. In women, an increase in *Negative self-image* score was significantly associated with lower odds of having shared one’s HIV status with no one or only 1–2 people (aOR 0.76 [95%CI 0.59–0.98]).

Higher scores in nearly all stigma domains were significantly associated with having 0–2 compared to having ≥ 3 close friends, for both men (e.g., *Concerns about public attitudes*: aOR 1.24 [95% CI 1.11–1.38]) and women (e.g., *Concerns about sharing status*: aOR 1.50 [95% CI 1.20–1.87]) (Table 5). A similar pattern was observed in relation to feelings of loneliness. Additionally, higher stigma scores across all domains were significantly associated with symptoms of anxiety in both men and women (e.g., *Negative self-image* in men: aOR 1.52 [95%CI 1.35–1.70]; in women: aOR 1.62 [95%CI 1.28–2.04]). Symptoms of depression, however, were only significantly associated with higher levels of stigma in men (e.g., *Concerns about public attitudes*: aOR 1.39 [95%CI 1.22–1.58]). Higher scores across all stigma domains were also associated with greater odds of reporting a history of suicidal thoughts or attempts in men. Among women, this association was only statistically significant in the *Negative self-image* domain (aOR 1.25 [95%CI 1.02–1.53]).

**Table 5:** Associations between HIV-related stigma and psychosocial characteristics among people with HIV in Denmark (*SHARE* study, 2021–2022)

	Social network		Loneliness		Anxiety		Depression		Suicidal tendencies	
	Number of close friends < 3 vs. ≥ 3 (REF)		Feeling lonely Sometimes, often or always vs. never or rarely (REF)		Symptoms in the last two weeks GAD-7 score ≥ 5 vs. < 5 (REF)		Symptoms in the last two weeks PHQ-2 score ≥ 3 vs. < 3 (REF)		Suicidal thoughts or attempts Ever vs. never (REF)	
	aOR^a^ [95% CI]		aOR^a^ [95% CI]		aOR^a^ [95% CI]		aOR^a^ [95% CI]		aOR^a^ [95% CI]	
	Men (*n* = 459)	Women (*n* = 130)	Men (*n* = 461)	Women (*n* = 134)	Men (*n* = 461)	Women (*n* = 135)	Men (*n* = 461)	Women (*n* = 135)	Men (*n* = 451)	Women (*n* = 132)
Total HSS-12 score	1.10 [1.06;1.14]	1.10 [1.02;1.16]	1.13 [1.09;1.17]	1.09 [1.03;1.15]	1.16 [1.11;1.21]	1.19 [1.10;1.30]	1.12 [1.07;1.17]	1.04 [0.98;1.11]	1.09 [1.05;1.12]	1.05 [0.98;1.12]
Personalised stigma	1.35 [1.20;1.52]	1.24 [1.03;1.49]	1.37 [1.22;1.53]	1.28 [1.06;1.54]	1.38 [1.22;1.55]	1.47 [1.18;1.84]	1.23 [1.09;1.39]	1.04 [0.84;1.28]	1.29 [1.15;1.44]	1.14 [0.92;1.42]
Concerns about sharing status	1.26 [1.14;1.40]	1.50 [1.20;1.87]	1.25 [1.13;1.37]	1.16 [0.98;1.37]	1.27 [1.15;1.41]	1.33 [1.07;1.64]	1.27 [1.12;1.43]	1.09 [0.88;1.34]	1.11 [1.02;1.22]	1.04 [0.85;1.26]
Concerns about public attitudes	1.24 [1.11;1.38]	1.29 [1.06;1.57]	1.38 [1.23;1.54]	1.16 [0.97;1.39]	1.52 [1.34;1.72]	1.51 [1.19;1.92]	1.39 [1.22;1.58]	1.09 [0.88;1.36]	1.30 [1.17;1.45]	1.11 [0.89;1.38]
Negative self-image	1.19 [1.07;1.31]	1.13 [0.96;1.32]	1.37 [1.24;1.52]	1.29 [1.09;1.53]	1.52 [1.35;1.70]	1.62 [1.28;2.04]	1.36 [1.21;1.53]	1.19 [0.99;1.44]	1.28 [1.15;1.41]	1.25 [1.02;1.53]

Reference group (REF); Adjusted odds ratio (aOR); Confidence intervals (CI); The 7-item Generalized Anxiety Disorder Screener (GAD-7); The 2-item Patient Health Questionnaire (PHQ-2); The 12-item short version of The HIV Stigma Scale (HSS-12). *Interpretation*: The aOR reflects the change in odds of the examined psychosocial characteristics per one unit increase in the HSS-12 score

Missing or ‘I do not know’ responses are not included in the aOR calculations, hence the numbers do not add up to 486 for men and 144 for women

^a^Adjusted for age, sexual identity, country of birth, relationship status, financial difficulties, history of mental health problems, time since HIV diagnosis and recreational drug use

As seen in Table 6, higher total stigma scores were statistically significantly associated with reduced sexual desire in the past year, in both men (aOR 1.09 [95%CI 1.05–1.13]) and women (aOR 1.12 [95%CI 1.04–1.20]), as well as with increased odds of erectile dysfunction in the last four weeks among men (aOR 1.04 [95%CI 1.00-1.08]). The *Negative self-image* domain was significantly associated with symptoms of female sexual dysfunction (aOR 1.45 [95%CI 1.03–2.05]) among women in relationships who had had sex in the past four weeks.

**Table 6:** Associations between HIV-related stigma and sexual health characteristics among people with HIV in Denmark (*SHARE* study, 2021–2022)

	Sexual desire		Sexual inactivity		Sexual dysfunction		Chemsex
	Felt a desire to have sex in the last year Less than once a month vs. at least once a month (REF)		Sex with another person in the last year No vs. yes (REF)		Symptoms of erectile dysfunction in the last four weeks IIEF-5^a^ scores 5–21 vs. 22–25 (REF)	Symptoms of sexual dysfunction in the last four weeks FSFI-6^a^ scores 6–19 vs. 20–30 (REF)	History of chemsex use^b^ Ever vs. never (REF)
	aOR^c^ [95% CI]		aOR^c^ [95% CI]		aOR^c^ [95% CI]	aOR^c^ [95% CI]	aOR^d^ [95% CI]
	Men (*n* = 439)	Women (*n* = 115)	Men (*n* = 436)	Women (*n* = 120)	Men (*n* = 243)	Women (*n* = 60)	Men (*n* = 107)
Total HSS-12 score	1.09 [1.05;1.13]	1.12 [1.04;1.20]	1.03 [0.99;1.06]	1.07 [0.98;1.17]	1.04 [1.00;1.08]	1.05 [0.96;1.16]	0.98 [0.91;1.05]
Personalised stigma	0.97 [0.80;1.18]	1.00 [0.83;1.20]	1.03 [0.91;1.17]	1.16 [0.86;1.56]	1.10 [0.96;1.27]	1.17 [0.90;1.53]	1.05 [0.83;1.34]
Concerns about sharing status	0.99 [0.86;1.14]	1.07 [0.90;1.27]	1.08 [0.98;1.19]	1.09 [0.84;1.41]	1.06 [0.95;1.18]	0.90 [0.69;1.18]	0.90 [0.73;1.10]
Concerns about public attitudes	1.06 [0.88;1.27]	1.00 [0.82;1.21]	1.11 [0.99;1.25]	1.29 [0.95;1.75]	1.12 [0.98;1.27]	1.13 [0.85;1.51]	0.94 [0.76;1.17]
Negative self-image	1.02 [0.86;1.22]	0.97 [0.82;1.14]	1.07 [0.96;1.19]	1.25 [0.97;1.61]	1.14 [0.99;1.30]	1.45 [1.03;2.05]	0.95 [0.78;1.15]

Standard deviations (SD); Odds ratio (OR); Confidence intervals (CI); The 5-item International Index of Erectile Function (IIEF-5); The 6-item Female Sexual Function Index (FSFI-6); The 12-item short version of The HIV Stigma Scale (HSS-12). *Interpretation*: The aOR reflects the change in odds of the examined sexual health characteristics per one unit increase in the HSS-12 score. Missing or ‘I do not know’ responses are not included in the aOR calculations, hence the numbers do not add up to 486 for men and 144 for women

^a^Only shown to respondents with a partner/spouse. Responses are based on sexual experiences in the last four weeks

^b^The question “Have you ever used drugs (other than alcohol or prescription medication) to enhance sexual desire, intensity or performance in relation to sex?” was put to men who reported having sex with men. The drugs include cocaine, gamma-hydroxybutyrate, methamphetamine, mephedrone ketamine, methylenedioxymethamphetamine (MDMA), amphetamine or other unspecified drugs

^c^Adjusted for age, sexual identity, country of birth, relationship status, financial difficulties, history of mental health problems, time since HIV diagnosis and recreational drug use

^d^Adjusted for age, country of birth, relationship status, financial difficulties, history of mental health problems and time since HIV diagnosis

## Discussion

This study investigated the associations between sociodemographic, health, lifestyle-related characteristics and stigma, as well as the potential link between stigma and HIV-specific characteristics and some previously identified disparities in psychosocial and sexual health among PWH. It was, to our knowledge, the first to do so in Denmark. We found that anticipated stigma, represented by the domains *Concerns about public attitudes* and *Concerns about sharing status*, was the highest scoring stigma mechanism, and that stigma was closely associated with nearly all the psychosocial characteristics and some of the sexual ones.

Recently published studies from Sweden [48], Switzerland [49] and Kenya [50], also employing the HSS-12 scale, have reported scores similar to ours. For example, in the domain *Concerns about public attitudes*, the Swedish study [48] reported a mean score of 7.7, the Swiss study [49] reported 7.2, and the Kenyan study [50] reported 8.5. Note that these data were not stratified by sex. Likewise, in *Personalised stigma*, the means were 6.2, 4.8 and 4.9, respectively, in the same order of studies. Interestingly, the order of the domains according to mean scores were also identical, indicating that based on these findings, anticipated stigma may pose the main stigma challenge to PWH living in Scandinavia, Europe and on the African continent, while enacted stigma appears to pose the lowest concern.

Originating from a low- or middle-income country was associated with higher stigma across most domains in our Danish study, consistent with previous findings based on global data that PWH of Black or Asian ethnicity report greater stigma than white PWH [14, 49], and that stigma related to HIV status is particularly high among migrants [51]. These findings underscore the risk of intersectional stigma among racial and ethnic minorities, highlighting the need for healthcare professionals to recognise the cumulative impact of multiple stigmas in PWH [14, 52]. These findings align with the minority stress theory, highlighting how stigma targeting marginalised identities, such as ethnicity or sexual minority status, can intensify psychosocial stress and contribute to poorer mental and sexual health outcomes [20]. A history of mental health problems was associated with higher stigma levels across nearly all stigma domains in our study, which accords well with findings from other countries [8, 15–17]. Furthermore, we found that a longer time since HIV diagnosis was significantly associated with lower levels of stigma, which also aligns with findings from other recent studies [16, 48, 49]. The time allowed to adjust to the diagnosis and develop coping strategies for stigma over time has been suggested as a possible explanation for this association [48]. Sexual identity, specifically identifying as something other than heterosexual, was associated with lower levels of stigma in two stigma domains. The existing literature does not show a clear consensus on this association. A meta-analysis concluded that sexual minority status is generally associated with higher stigma levels, although this conclusion was primarily based on studies from the United States [14]. In contrast, recent data from Switzerland, also using the HSS-12 scale, showed that heterosexual PWH had higher stigma levels than non-heterosexuals [49]. Such variability highlights the necessity of understanding intersectional stigma within specific cultural and structural contexts [21–23].

In men, all stigma domains were significantly associated with higher odds of HIV status sharing with no one or only 1–2 people. In women, however, only one significant association was found: higher levels of stigma in the *Negative self-image* domain, representing internalised stigma, were linked to lower odds of having shared one’s status with less than three people. Despite examining our data for possible interactions and other explanations, this finding remains unexplained. Empirically, we have not found any studies conducted among women with HIV that specifically examine associations between internalised stigma and status sharing to help explain this finding.

Keeping one’s medical information private can serve as a form of self-protection, especially among ethnic minorities concerning HIV status [51]. However, research suggests that HIV status sharing is linked to better health outcomes and reduced social isolation [15] and has been shown to mitigate the effects of stigma among PWH [48, 53]. Unlike other researchers [7, 18, 19], we observed no significant associations between stigma and HIV self-management. This might be a statistical power issue due to the very limited number of individuals with suboptimal management in our study, reflecting that study participants were actively recruited in medical settings.

Higher levels of stigma were associated with having only few close friends and with feelings of loneliness among both men and women. Stigma – particularly anticipated stigma, the highest scoring stigma domains in our study – is well recognised to be associated with reduced social support, often driven by fear of HIV-related rejection when engaging in social connections [13, 33]. This could potentially create a self-perpetuating cycle, where stigma leads to social withdrawal, which in turn reinforces stigma. Social support has been shown to alleviate the stress of stigma [15], and, therefore, has the potential to break this cycle, fostering greater social engagement. Anxiety, depression and suicidal tendencies have all been linked to higher levels of stigma in previous research [15, 16] among both men and women, a trend also reflected in our findings. Depression and suicidal tendencies were, however, only statistically significantly associated with stigma among male participants in our study.

Other studies have linked stigma with affected sexual health in both men and women with HIV [54, 55]. We included several sexual health characteristics that we previously identified as being associated with living with HIV in Denmark [26–28]. Stigma was significantly linked to reduced sexual desire in both men and women. Additionally, stigma was significantly associated with female sexual dysfunction and, with a borderline significant association, with erectile dysfunction.

### Strengths and Limitations

This exploratory study is the first to provide insights into HIV-related stigma and its associations with psychosocial and sexual health among PWH in a Danish context. A key strength of the study is its use of validated and standardised patient-reported outcome measures, as recommended for cross-sectional studies [56] enhancing the reliability of the data. The main limitation is its cross-sectional design preventing causal inferences regarding the observed associations between stigma and the HIV-specific, psychosocial and sexual characteristics examined. Another limitation to consider is the recruitment method; participants were recruited by healthcare professionals, which may impact the findings related to HIV self-management, as participants were likely already engaging in care. Furthermore, our results are based on a relatively small sample of the target population of PWH in Denmark (~ 10%). It is reasonable to assume that individuals with fewer physical or mental resources may be underrepresented in our study population, which could affect the generalisability of our findings. Additionally, the smaller number of women in our sample reduced the statistical power to detect associations in this group. As with all observational studies, residual confounding may also be present.

## Conclusion

In conclusion, this study highlights that anticipated stigma, especially concerns related to HIV status sharing, is the predominant aspect of stigma experienced by PWH in Denmark. Our finding that HIV-related stigma is positively linked to being born in a low- or middle-income country, to having shared one’s HIV status with no one or only one or two people and to a range of other unfavourable psychosocial and sexual characteristics highlight the need for interventions to mitigate anticipated stigma among PWH in Denmark. Such interventions could include public awareness campaigns to reduce HIV-related misconceptions, educational programs or targeted efforts to normalise discussions around HIV status in healthcare and community settings. Additionally, future studies and interventions should incorporate intersectional stigma and minority stress theory to better understand how overlapping marginalised identities and other underlying factors sustain stigma among well-treated PWH, even in high-resource settings, despite advancements in treatment and care.

## Electronic Supplementary Material

Below is the link to the electronic supplementary material.

Supplementary Material 1

## Data Availability

The study data are protected in compliance with Danish data protection guidelines and are not readily available.

## Code Availability

Codes are available upon request.
