# Supplementary material for: HIV-related Stigma among People with HIV in Denmark and its Association with Psychosocial and Sexual Health: a cross-sectional Nationwide Study
Source: AIDS Behav. 2025 Jun 14;29(11):3646–60. doi: 10.1007/s10461-025-04806-8 (PMC12500774; doi:10.1007/s10461-025-04806-8)
Supplement: Supplementary file 1 — Supplementary Material 1 [file 10461_2025_4806_MOESM1_ESM.docx]

**HIV-related stigma among people with HIV in Denmark and its association with psychosocial and sexual health: a cross-sectional nationwide study**

Supplementary Materials

| **Table S1: Univariate linear regression analysis of factors associated with HIV-related stigma among people with HIV in Denmark (*SHARE* study, 2021-2022)** | | | | | | | | | | | | |
| --- | --- | --- | --- | --- | --- | --- | --- | --- | --- | --- | --- | --- |
|  | |  | **Total HSS-12 score** | | **Personalised stigma** | | **Concerns about sharing HIV status** | | **Concerns about public attitudes** | | **Negative self-image** | |
|  |  | n (%) | β | [95% CI] | β | [95% CI] | β | [95% CI] | β | [95% CI] | β | [95% CI] |
| **Sex** | |  |  |  |  |  |  |  |  |  |  |  |
|  | Man | 486 (74.1) | REF |  | REF |  | REF |  | REF | REF | REF | REF |
|  | Woman | 144 (22.9) | **2.693** | **[1.354; 4.032]** | **0.874** | **[0.490; 1.259]** | **0.480** | **[0.010; 0.950]** | **0.653** | **[0.247; 1.058]** | **0.686** | **[0.255; 1.117]** |
| **Age** | |  |  |  |  |  |  |  |  |  |  |  |
|  | Per 1-year increase | 630 (100) | **-0.106** | **[-0.155; -0.057]** | -0.008 | [-0.022; 0.006] | **-0.027** | **[-0.044; -0.009]** | **-0.039** | **[-0.053; -0.024]** | **-0.033** | **[-0.049; -0.017]** |
|  | 18-39 years | 72 (11.4) | REF |  | REF |  | REF |  | REF | REF | REF | REF |
|  | 40-59 years | 378 (60.0) | -1.570 | [-3.389; 0.249] | 0.196 | [-0.333; 0.724] | -0.259 | [-0.895; 0.378] | **-0.829** | **[-1.373; -0.284]** | **-0.679** | **[-1.260; -0.097]** |
|  | ≥60 years | 180 (28.6) | **-3.439** | **[-5.411; -1.467]** | -0.050 | [-0.623; 0.523] | **-0.753** | **[-1.443; -0.063]** | **-1.358** | **[-1.949; -0.768]** | **-1.278** | **[-1.908; -0.647]** |
| **Country of birth^a^** | |  |  |  |  |  |  |  |  |  |  |  |
|  | Born in Denmark or other high-income country | 525 (83.5) | REF |  | REF |  | REF |  | REF | REF | REF | REF |
|  | Born in low- or middle-income country | 104 (16.5) | **3.823** | **[2.318; 5.328]** | **0.973** | **[0.538; 1.408]** | **1.013** | **[0.486; 1.541]** | **0.957** | **[0.501; 1.413]** | **0.880** | **[0.393; 1.366]** |
| **Relationship status** | |  |  |  |  |  |  |  |  |  |  |  |
|  | Married/in steady relationship | 379 (61.4) | REF |  | REF |  | REF |  | REF | REF | REF | REF |
|  | Single | 238 (38.6) | 0.504 | [-0.681; 1.689] | 0.060 | [-0.281; 0.401] | -0.125 | [-0.536; 0.287] | 0.150 | [-0.207; 0.506] | **0.419** | **[0.041; 0.798]** |
| **Sexual identity** | |  |  |  |  |  |  |  |  |  |  |  |
|  | Heterosexual | 231 (36.7) | REF |  | REF |  | REF |  | REF | REF | REF | REF |
|  | Non-heterosexual | 399 (63.3) | **-0.572** | **[-0.911; -0.232]** | **-0.572** | **[-0.911; -0.232]** | **-0.635** | **[-1.046; -0.223]** | **-0.534** | **[-0.890; -0.179]** | -0.334 | [-0.715; 0.046] |
| **Employment status** | |  |  |  |  |  |  |  |  |  |  |  |
|  | Employed/full-time student | 427 (67.8) | REF |  | REF |  | REF |  | REF | REF | REF | REF |
|  | Unemployed or other^b^ | 203 (32.2) | 0.136 | [-0.214; 0.487] | 0.136 | [-0.214; 0.487] | **-0.891** | **[-1.309; -0.473]** | **-0.473** | **[-0.838; -0.108]** | -0.354 | [-0.743; 0.036] |
| **Education** | |  |  |  |  |  |  |  |  |  |  |  |
|  | Medium or long (>10 years) | 541 (86.3) | REF |  | REF |  | REF |  | REF | REF | REF | REF |
|  | Short (≤10 years) | 86 (13.7) | 1.412 | [-0.246;3.071] | **0.555** | **[0.078; 1.031]** | 0.127 | [-0.450; 0.705] | 0.173 | [-0.326; 0.673] | **0.557** | **[0.026; 1.088]** |
| **Financial difficulties**^c^ | |  |  |  |  |  |  |  |  |  |  |  |
|  | No | 542 (87.6) | REF |  | REF |  | REF |  | REF | REF | REF | REF |
|  | Yes | 77 (12.4) | 1.352 | [-0.291; 2.996] | 0.465 | [-0.008; 0.939] | -0.227 | [-0.799; 0.345] | 0.407 | [-0.089; 0.902] | **0.708** | **[0.182; 1.233]** |
| **History of physical health problems**^d^ | |  |  |  |  |  |  |  |  |  |  |  |
|  | No | 401 (64.9) | REF |  | REF |  | REF |  | REF |  | REF |  |
|  | Yes | 217 (35.1) | 0.402 | [-0.797;1.602] | 0.233 | [-0.110;0.576] | -0.267 | [-0.684;0.151] | 0.221 | [-0.140;0.582] | 0.215 | [-0.170;0.600] |
| **History of mental health problems**^e^ | |  |  |  |  |  |  |  |  |  |  |  |
|  | No | 382 (61.3) | REF |  | REF |  | REF |  | REF | REF | REF | REF |
|  | Yes | 241 (38.7) | **1.442** | **[0.271; 2.612]** | **0.359** | **[0.021; 0.696]** | -0.106 | [-0.516; 0.304] | **0.532** | **[0.180; 0.884]** | **0.657** | **[0.283; 1.030]** |
| **Current smoker** | |  |  |  |  |  |  |  |  |  |  |  |
|  | No | 494 (78.7) | REF |  | REF |  | REF |  | REF | REF | REF | REF |
|  | Yes | 134 (21.3) | -1.246 | [-2.635; 0.144] | -0.270 | [-0.670; 0.130] | **-0.785** | **[-1.264; -0.306]** | 0.385 | [-0.803; 0.034] | 0.195 | [-0.251; 0.641] |
| **Alcohol intake** | |  |  |  |  |  |  |  |  |  |  |  |
|  | <7 units per week | 460 (74.6) | REF |  | REF |  | REF |  | REF | REF | REF | REF |
|  | ≥7 units per week | 157 (25.4) | -0.092 | [-1.403; 1.220] | **-0.383** | **[-0.760; -0.007]** | 0.128 | [-0.329; 0.584] | 0.191 | [-0.204; 0.586] | -0.027 | [-0.449; 0.394] |
| **Recreational drug use**^f^ | |  |  |  |  |  |  |  |  |  |  |  |
|  | Never | 299 (48.1) | REF |  | REF |  | REF | REF | REF | REF | REF | REF |
|  | Ever | 323 (51.9) | -1.096 | [-2.227; 0.036] | **-0.463** | **[-0.785; -0.142]** | **-0.533** | **[-0.929; -0.137]** | -0.054 | [-0.397; 0.289] | -0.045 | [-0.411; 0.320] |
| **Time since HIV diagnosis** | |  |  |  |  |  |  |  |  |  |  |  |
|  | Per 1-year increase | 630 (100) | **-0.125** | **[-0.184; -0.066]** | -0.005 | [-0.022; 0.012] | **-0.052** | **[-0.072; -0.032]** | **-0.032** | **[-0.050; -0.015]** | **-0.035** | **[-0.054; -0.016]** |
| *Note*: The 12-item short version of The HIV Stigma Scale (HSS-12). *Interpretation*: The β value reflects the change in HSS-12 score for each category, or per 1-year increase, of the covariate compared to its reference group. Numbers do not always sum up to 630 as missing or "I do not know" responses were excluded from the analyses. | | | | | | | | | | | | |
| ^a^ Indexed according to The World Bank world development indicators. | | | | | | | | | | | | |
| ^b^ Includes unemployed, long-term sick leave, retirement or disability-related early retirement, parental leave and other unspecified statuses. | | | | | | | | | | | | |
| ^c^ Answers to the question: “In the last year, have you had any difficulty paying your bills?” | | | | | | | | | | | | |
| ^d^ Answers to the question “Have you ever been treated by a doctor for a long-lasting or severe physical disease (other than HIV)?” | | | | | | | | | | | | |
| ^e^ Answers to the question “Have you ever received treatment by a doctor, psychologist or similar professional for a mental health problem?” | | | | | | | | | | | | |
| ^f^ Includes cannabis, euphoriant or hallucinogenic drugs. | | | | | | | | | | | | |
